# Discriminating orientation information with phase consistency in alpha and low-gamma frequency bands: an EEG study

**DOI:** 10.1038/s41598-024-62934-y

**Published:** 2024-05-25

**Authors:** Alireza Khadir, Shamim Sasani Ghamsari, Samaneh Badri, Borhan Beigzadeh

**Affiliations:** Biomechatronics and Cognitive Engineering Research Lab, School of Mechanical Engineering, Iran University of Science and Technology, Tehran, Iran

**Keywords:** Cognitive neuroscience, Working memory

## Abstract

Recent studies suggest that noninvasive imaging methods (EEG, MEG) in the human brain scalp can decode the content of visual features information (orientation, color, motion, etc.) in Visual-Working Memory (VWM). Previous work demonstrated that with the sustained low-frequency Event-Related Potential (ERP under 6 Hz) of scalp EEG distributions, it is possible to accurately decode the content of orientation information in VWM during the delay interval. In addition, previous studies showed that the raw data captured by a combination of the occi-parietal electrodes could be used to decode the orientation. However, it is unclear whether the orientation information is available in other frequency bands (higher than 6 Hz) or whether this information is feasible with fewer electrodes. Furthermore, the exploration of orientation information in the phase values of the signal has not been well-addressed. In this study, we propose that orientation information is also accessible through the phase consistency of the occipital region in the alpha band frequency. Our results reveal a significant difference between orientations within 200 ms after stimulus offset in early visual sensory processing, with no apparent effect in power and Event-Related Oscillation (ERO) during this period. Additionally, in later periods (420–500 ms after stimulus offset), a noticeable difference is observed in the phase consistency of low gamma-band activity in the occipital area. Importantly, our findings suggest that phase consistency between trials of the orientation feature in the occipital alpha and low gamma-band can serve as a measure to obtain orientation information in VWM. Furthermore, the study demonstrates that phase consistency in the alpha and low gamma band can reflect the distribution of orientation-selective neuron numbers in the four main orientations in the occipital area.

## Introduction

It is widely believed that visual information such as color^1–5^, motion^6,7^, and orientation^8–12^ are being maintained in Visual-Working-Memory (VWM). Recent studies provide evidence that orientation decoding with noninvasive electrophysiological methods is possible. For example, Harrison and Tong^13^ used functional magnetic resonance imaging (fMRI) and pattern classification methods. They showed that even at low overall activity levels, orientations maintained in VWM could be decoded from activity patterns in the human visual cortex (specifically in V1–V4 areas). Moreover, Multivariate pattern analysis (MVPA) of fMRI data can also successfully decode the orientation of a visual stimulus in the human visual cortex^14–17^. Cichy et al.^18^ used magnetoencephalography (MEG) experiments and found that at around 53-ms after stimulus onset (with a 95 percent confidence interval of 50–61-ms), the signals contained information about the orientation of stimuli that peaked at 103-ms (91–153-ms ). In contrast, with an analogous decoding procedure, they could not reveal any phase information from the MEG signals.

Subsequently, a comparison between cardinal and oblique orientation stimuli is discussed in the literature. For example, with MEG recording, Pantazis et al.^19^ found that multivariate methods are well suited for analyzing gamma oscillations, and can robustly encode orientation information, in addition to predominantly discriminating cardinal from oblique stimuli. Their decoding method showed that two dominant frequency bands at 24–32 Hz and 50–58 Hz have higher decoding accuracy for cardinal vs. oblique orientations. Similar results were also obtained for evoked MEG responses. Indeed, whether the cardinal or oblique stimuli have a stronger response depends on the type of stimulus. For instance, Maloney et al.^20^ analyzed fMRI data using the MVP approach and found that the V1 response was maximal for vertical orientations at low stimulus contrast, whereas high stimulus contrast showed that the V1 response was maximal for the oblique orientations.

Furthermore, many recent studies have demonstrated that the electroencephalogram (EEG) activity of scalp distribution could also be used to decode orientation information. Bae and Luck^21^ used the scalp distribution of low-frequency Event-Related-Potential (ERP under 6 Hz) and alpha-band power (8–12 Hz) to decode the specific orientation value being maintained in VWM. They showed that during the retention period, it is possible to determine the exact location-independent orientation of the to-be-remembered item with sustained ERP activity. Moreover, they predicted that alpha-band oscillations would reflect the spatial location of the object rather than the contents of VWM. This result was consistent with the previous research that decoded the location being maintained in spatial VWM with alpha-band activity (Foster et al.^11,22^). Yet, there was a lack of information about the specific brain areas and their patterns of activation mechanism in terms of band frequency, latency, and phase coherency. In another study, Hajonides et al.^5^ demonstrated that orientation decoding from scalp distribution reflects sensory qualities in significantly activated posterior electrodes contralateral to the presented stimulus. However, they focused mostly on decoding methods, which are reliable for decoding both color and the stimuli’s orientation. And there is a lack of sufficient evidence about the time-frequency-dependent mechanism.

Nevertheless, these findings and the available literature focused mostly on the decoding method and used low-frequency ERP or power-band analysis. Therefore, the critical question remains whether there are any specific brain areas in which the maintained orientation information in VWM is distributed. And whether particular frequency-bands can carry orientation information. Additionally, is there any other effective measurement, such as phase, that could be used whenever the power and ERP provide insufficient information.

Therefore, our major aim in this study was to address these questions. We used a task in which participants were presented with four different Gabor gratings, each containing only the orientation information. We, therefore, shifted our focus from the decoding procedure, lowered the resolution, and used only 16 electrodes to find the time-frequency-dependent mechanism of orientation representation in VWM. Our experimental design results indicated a significant difference in the phase consistency value of different orientations in the presented stimulus, both in alpha and low-gamma-band frequencies. Therefore, it suggests that in addition to ERP and Power analysis, Inter-Trial-Phase-Consistency (ITPC) could also be an effective measurement to decode the specific orientation of the presented stimuli maintained in VWM.

## Methods

The present study has been approved by the Iran University of science and technology. All the applied procedures conform to the Declaration of Helsinki (1964)^23^ of the World Medical Association concerning human experimentation.

### Experimental set-up

Nineteen male students aged 19–27 years old and no previous experiment participated in this study. All subjects provided informed consent in accordance with the local Ethics committee. There were no reports of a particular illness, and they all had a normal or correct-to-normal vision. Participants sat in a dark room, isolated acoustically and electrically, in front of a CRT monitor with a resolution of 1024 $\times$ 768 pixels and a 60 Hz refresh rate at a distance of approximately 60 cm. They were told to avoid movements and blinking during this procedure. Four participants were excluded during pre-processing due to excessive eye movements, and further, the data of 15 participants were examined in the analysis.

### Apparatus and stimuli

**Figure 1:**
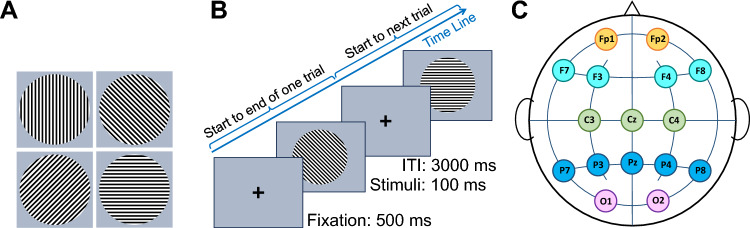
Stimulus characteristics and presentation. (**A**) Four different orientations of luminance-defined sinusoidal Gabor gratings (0^∘^, 45^∘^, 90^∘^, 135^∘^, spatial frequency = 0.1 Hz, phase = 0^∘^) presented with Black and White colors on a gray background. (**B**) The visual paradigm consists of 24 randomly selected stimuli, starting with a 500 ms fixation point, after which participants were presented with one Gabor gratings for 100 ms followed by a 3000 ms delay period of fixation. (**C**) The location of the electrodes is based on the 10–20 system, and the areas marked on the electrodes are the regions mentioned in this paper. The occipital area includes O1 and O2 electrodes that are marked with the purple; the parietal region includes Pz, P3, P4, P7, P8 (all marked with blue), the central region includes Cz, C3, C4 (marked with green); frontal area (F3, F4, F7, F8) marked with cyan, and prefrontal area (Fp1, Fp2) marked with orange.

Stimuli were generated in MATLAB 2017b (MathWorks, Natick, MA^24^) using Psychophysics Toolbox Version 3.0.11^25^. The stimuli consisted of luminance-defined sinusoidal Gabor gratings. Gratings were presented using four different orientations (0^∘^, 45^∘^, 90^∘^, 135^∘^, spatial frequency = 0.1 Hz, phase = 0^∘^), with Black and White colors on a gray background (see Fig. 1A).

Each Block contained 24 randomly selected stimuli (six times repetition of each stimulus) presented for 100-ms with a delay period of 3 sec (Fig. 1B). This Block was presented five times for each subject; therefore, we have an average of 30 trials of each orientation.

### EEG recording and preprocessing

The EEG data were recorded from 16 Ag/AgCl electrodes mounted in an elastic cap (ScienceBeam eWave system) at scalp sites locations of Fp2, Fp1, F7, F3, F4, F8, C4, Cz, C3, P7, P3, Pz, P4, P8, O1, and O2. The left and right mastoids attached electrodes used as reference and ground sites. EEG data were recorded at a sampling rate of 1000 Hz. Custom-written MATLAB scripts and commands from the EEGLAB toolbox^26^ were used for all EEG preprocessing and processing. Trials where participants blinked at the time of stimulus onset and trials with high within-trial variance were removed before further analysis.

All EEG raw data were filtered with a highpass filter at 0.5 Hz by using ”eegfilt” in the EEGLAB toolbox. In order to effectively remove available edge artifacts, 2000-ms prior to and 2000-ms after the stimulus onset were epoched. To remove artifacts from the data, we used FASTER (Fully Automated Statistical Thresholding for EEG artifact Rejection^27^) algorithm, which uses a statistical threshold for reject bad channels and epochs. Note that after necessary filtration and Time-Frequency analysis, we excluded only $-500$ to 1500-ms relative to stimulus onset for further analysis. After preprocessing, we have, on average, a 15 (Subject) $\times$ 16 (Channel) $\times$ 2000 (sample point) $\times$ 30(trials) 4D matrix for each of the four orientations.

### ERO and time-frequency analysis

The raw EEG signal was bandpass filtered to capture only frequency-specific activity, using ”eegfilt()” from EEGLAB Toolbox, which uses a zero-phase FIR bandpass filter (with an order of $3*fix(Fs/lowcutoff)$). Next, we applied a Hilbert Transform from MATLAB Signal Processing Toolbox to the bandpass-filtered data. The Hilbert transform extracts a complex signal from a signal that contains only a real part, and it can be represented using Euler’s formula: $M(t)e^{i2\pi ft}$. The $M(t)$ is extracted as the instantaneous amplitude, and the following Matlab syntax is used to extract the complex analytic signal:

1 $$\begin{aligned} {hilbert(eegfilt(data, fs, lowcuto\, f\, f, highcuto\, f\, f)')'} \end{aligned}$$

where fs is the sampling frequency ($1000 Hz$), and we used delta$(1-4 Hz)$, theta $(4-8 Hz)$, alpha $(8-12 Hz)$, beta $(12-30 Hz)$, low gamma $(30-50 Hz)$, and high gamma $(70-150 Hz)$ for the lower and upper bound. The mean() of trials for each filtered frequency band was computed for the Event-related oscillation (ERO) analysis of their activity.

Inter-Trial Phase Clustering (ITPC) was used for the examination of cortical phase synchrony or for measuring the phase consistency over trials. ITPC measures the extent to which a distribution of phase angles at each time-frequency-electrode point across trials is nonuniformly distributed in polar space. The Mathematical representation of ITPC is as follows:

2 $$\begin{aligned} {ITPC_{tf} = n^{-1} \Sigma ^{n}_{r=1} e^{i K_{tfr}} } \end{aligned}$$

where n is the number of trials, $e^{ik}$ is taken from Euler’s formula and provides the complex polar representation of a phase angle $k$ on trial $r$ at time-frequency point $tf$. The MATLAB syntax for computing the $k$ is as follows:

3 $$\begin{aligned} {angle(hilbert(eegfilt((data, fs, lowcuto\,f\,f, highcuro\,f\,f) ')).} \end{aligned}$$

ITPC is bounded by 0 and 1, with 0 indicating random phases and 1 indicating perfect phase coherency. In addition, because of the Gaussian shape of the frequency response of Morlet wavelets, wavelet convolution tends to produce smooth-looking and, therefore, easily visually interpretable time-frequency plots (Mike X Cohen^28^). Only for an examination of the visual response of our study, we performed a fine-grained complex Morlet wavelet convolution to the EEG epochs to analyze the data in the time-frequency domain with frequencies ranging from 2 to 80 Hz in 80 logarithmically spaced steps. It is emphasized that we core analysis of our study is Hilbert transform for six frequency bands.

### Examination of brain oscillation

To determine the nature of observations within the power spectrum, including whether they exhibit true oscillatory characteristics, are associated with aperiodic activity, or result from ERP contamination in the power spectrum, we employed an advanced spectral parameterization approach known as ”fitting oscillations and one over f” (FOOOF) (Donoghue et al., 2020b). This algorithm facilitates the decomposition of the neural signal into its distinct periodic and aperiodic components. For each participant and orientation, the ERP component of each trial was initially subtracted, and subsequently, the Power Spectral Density (PSD) was computed using Welch’s method with a 2-second Hamming window and 50% overlap in Matlab. The FOOOF Python toolbox (version 1.0.0; https://fooof-tools.github.io/fooof/) was then employed to parameterize the spectral data, effectively separating the periodic and aperiodic components of the signal. Through this approach, PSDs were treated as a linear combination of both aperiodic activity and oscillatory peaks, with amplitudes extending beyond the aperiodic signal, as elucidated in prior literature^29,30^. This comprehensive methodology enabled a meticulous characterization of the neural signal, disentangling periodic and aperiodic contributions within the power spectrum.

### Topographical maps

To illustrate the different ERO and time-frequency measures of the scalp distributions in each time window of interest, we created topographical maps using spline interpolation of the power and ITPC value differences between the orientations. In our topographic map, the occipital region was demarcated by electrodes O1 and O2. The parietal region encompassed electrodes P8, P4, Pz, P3, and P7. Electrodes within the central region included P8, P4, Pz, P3, and P7. The frontal region was defined by electrodes F8, F4, F3, and F7. Lastly, the prefrontal region comprised electrodes Fp1 and Fp2; see Fig. 1C.

We utilized the Matplotlib toolbox^31^ in our analysis, opting for the ”viridis” colormap to ensure perceptually uniform representation and mitigate any potential distortion in the data. This choice deviates from the common usage of the ”jet” colormap, a decision made to enhance the accuracy and reliability of our visualizations^32^.

### Statistical analyses

To compare the ERO for each pair of orientations, we performed a two-sided Wilcoxon test for each time point, which does not make assumptions about the underlying distribution. We implemented a whole-subject permutation method to test the significance of ERO waveform in different orientations. Inverted random subsets of ERO values are used on each iteration (Chapter 13, Luck 2014^33^). For multiple comparison corrections over time, we used a cluster-based permutation analysis (Maris and Oostenveld, 200^34^) with 1,000 permutations at an evaluation threshold of 0.05 on the data. Cluster-based permutation analysis is a robust method known for its enhanced sensitivity, type I error control, flexibility, and interpretability. This approach was chosen due to its advantages in correcting for multiple comparisons in time series neural analysis.

This methodology was extended to compare ERO activity across multiple orientations using a Non-parametric Friedman Test, akin to a parametric repeated measures ANOVA. Subsequently, the Wilcoxon Signed-Rank test served as the post hoc analysis to discern specific differences between conditions. Effect sizes were computed using the formula $r=Z/\sqrt{N}$ (Rosenthal 1994)^35^, where values falling within the range of 0.10 to < 0.30 were characterized as small, 0.30 to 0.50 as medium, and $\ge$ 0.50 as large (Cohen 2013)^36^.

The same procedure was used for power, and ITPC values. To evaluate the statistical significance of the resulting time-frequency analysis, the above procedure was repeated for each time point and frequency. Furthermore, to evaluate the statistical significance of Topographical maps, we implemented the whole-subject permutation analysis with a two-sided Wilcoxon test used for 16 electrodes with n=5000 permutations and a cluster-forming significance threshold of p < 0.01. This comprehensive approach ensured rigorous correction for multiple comparisons and robust statistical assessment across various analyses within our study.

### Data-driven approach

We employed exploratory data-driven methodologies to identify significant differences in orientation information. To compare results across various stimulus orientations, ERO, power, and ITPC values for each channel were extracted using the Hilbert transform method within six distinct frequency bands (delta, theta, alpha, beta, low gamma, high gamma). The resulting data from 16 electrodes corresponding to a specific orientation were averaged to create distinct regions of interest, such as Occipital, Parietal, Occi-parietal, Central, Centro-parietal, Frontal, Centro-Frontal, Prefrontal, and Temporal. Such approach was similarly applied to our previous study^37^.

**Figure 2:**
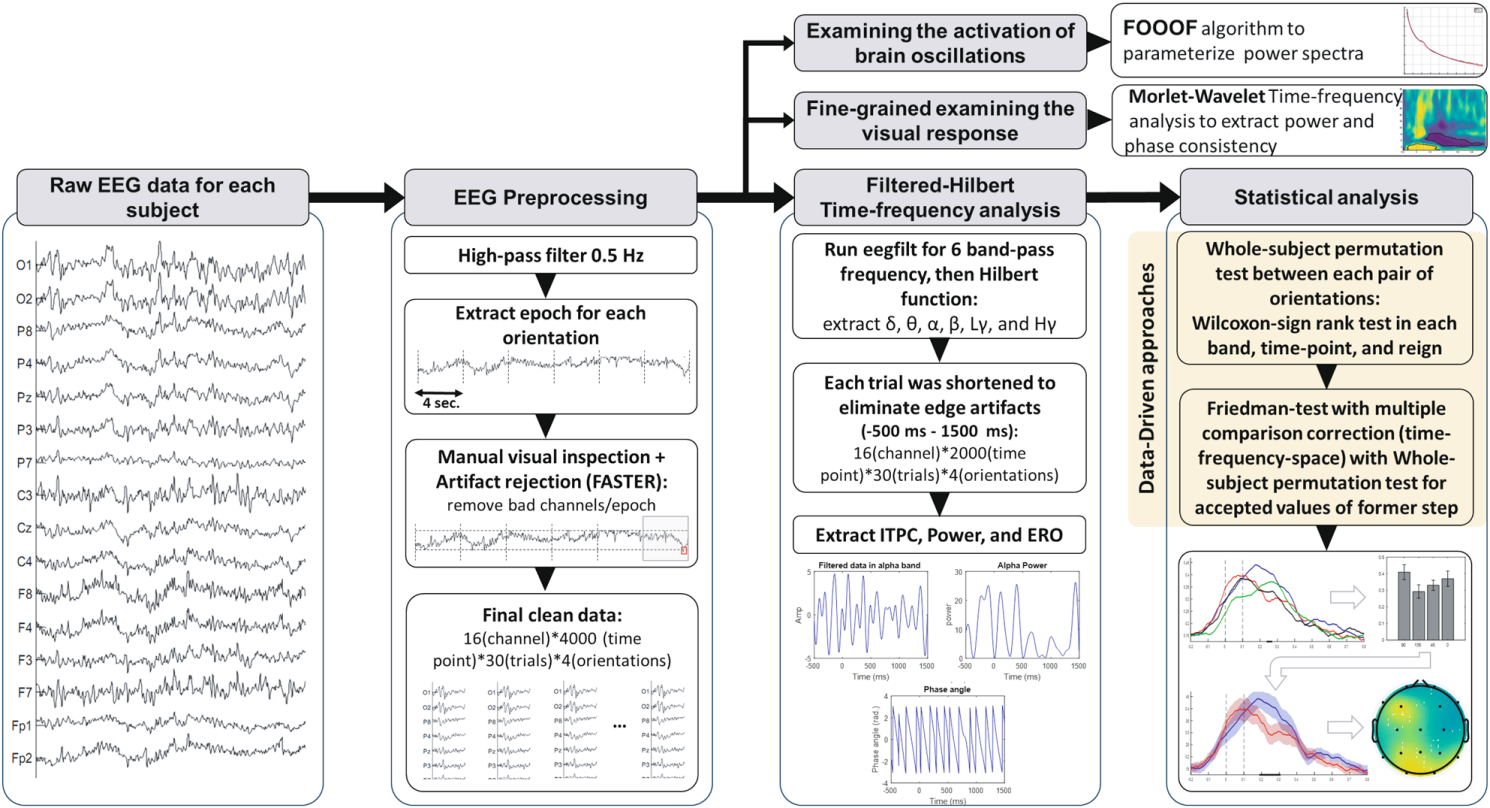
Flowchart of the procedure.

Subsequently, the Wilcoxon signed-rank test with cluster-based multiple comparisons correction, as detailed in the statistical section, was applied to each time point to investigate the significance of differences in brain areas and band frequencies concerning ERO components, power, and ITPC for each pair of stimuli. An exploratory analysis revealed two robust effects in the ITPC analysis of the occipital area within the alpha and low gamma frequency bands. Focusing on these spatial-frequency regions, the Friedman test was applied to compare orientations. Figures 4 and 5 illustrate the analysis of these two frequency bands in the occipital area, highlighting the salient findings of the study; Fig. 2 shows the details of the proceudre.

## Results

**Figure 3:**
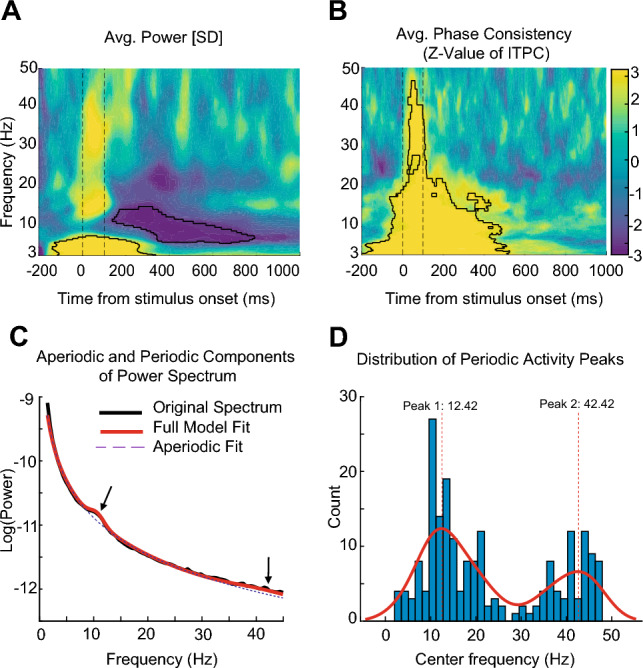
Time-Frequency decomposition of neural EEG responses to visual stimuli in occipital (O1 and O2) electrodes. Average time-frequency plots of 15 participants around stimulus onset using the Morlet Wavelets Convolution method. Plots are averaged over all orientation conditions. for (**A**) power analysis, values are normalized by baseline (z-score) of a 300–100 ms time-window before stimulus onset. In the map, yellow indicates that the power is higher than baseline periods, and blue indicates the opposite. The highlighted cluster in the delta & theta-frequency band (under 8 Hz) indicates a significant power increase immediately after stimuli onset, and in alpha-frequency band (8–12 Hz) indicates an evident power suppression during long periods after stimulus offset (permutation test with 1000 permutations, participant = 15, cluster-forming threshold p < 0.05, corrected significance level p < 0.001). Dash lines indicate the time of stimulus onset (t = 0 ms) and stimulus offset (t = 100 ms). (**B**) ITPC analysis, and for each frequency, values are subtracted from baseline periods (300–100 ms time-window before stimulus onset). The highlighted cluster showed a significant increase of under 25 Hz activity immediately after the presentation of the stimulus (permutation test with 1000 permutations, participant = 15, cluster-forming threshold p < 0.05, corrected significance level p < 0.001). (**C**) The power-spectral density represents the average across subjects and all conditions, and the FOOOF model is fitted to these average power spectra. Model parameters are extracted, with the indicated arrow highlighting the identified peaks. (**D**) The distribution illustrates all periodic peaks extracted from the FOOOF model for each subject and each condition. Notably, two prominent peaks are observed around 12 and 42 Hz.

The Time-Frequency decomposition of power activity in occipital sensors is depicted in Fig. 3A. The power response was obtained by normalizing the power of individual frequency bands to units of standard deviations (SDs) from baseline (Z-scored, a 300–100 ms window before stimulus onset). The result showed a significant increase in power activity of under 8 Hz immediately after stimulus onset and the suppression in the alpha range (8–12 Hz) around 200 ms after the presentation of the stimulus, which continued until 800 ms after stimulus onset. The observed suppression in the alpha frequency range aligns with existing reports indicating a consistent decrease in alpha-band activity during Working Memory tasks^38^. This phenomenon, characterized by a suppression of alpha oscillations, is well-documented in studies focusing on tasks that necessitate engagement with Working Memory processes. Importantly, this alpha suppression is considered indicative of the neural mechanisms underlying the formation and maintenance of active representations within the Working Memory system^39–41^. For statistical analysis we performed a Wilcoxon test (for further detail, see Methods), and cluster-based multiple comparison correction, the extended Whole-subject-permutation test with randomized sign change is used (1000 permutations, participant = 15, p < 0.001, corrected; cluster-forming threshold p < 0.05). Notably, at the lowest power frequencies, for example, under 8 Hz, the filtering procedure blurs the amplitude signal, which can result in low-frequency responses that apparently commence before stimulus onset.

Figure 3B demonstrates the Time-Frequency decomposition of the Z-value with the Wilcoxon test for phase consistency (ITPC) in occipital sensors (the value of ITPC is subtracted from the baseline activity of a 300-100 ms window before stimulus onset). The result showed a significant increase of under 25 Hz activity immediately after the presentation of the stimulus. To evaluate the statistically significant effect, we performed the same method as Fig. 3A.

To confirm the presence of brain oscillations in our study, we employed the FOOOF algorithm, as outlined in the methods section. For every participant and orientation, the ERP component of each trial was subtracted initially. Subsequently, the Power Spectral Density (PSD) was computed using Welch’s method, within a time window extending from 0 to 1 second after stimulus onset. This approach allowed us to systematically investigate the spectral characteristics of the neural activity, capturing relevant oscillatory patterns during the specified time interval.

The FOOOF model was used in the fixed mode (no spectral knee) with peak width limits = [0.5,20], maximum number of peaks = 15, peak threshold = 2, minimum peak height = 0.15. Power spectra were parameterized across the frequency range 1–55 Hz. As we can see in Fig. 3C, the good model fits for the FOOOF algorithm were observed (R2 =0.998, Error = 0.018). The FOOOF model identified two distinct peaks in the Power Spectral Density (PSD). The primary peak exhibits a center frequency of 10.41 Hz, an amplitude of 0.13 Hz, and a bandwidth of 1.26 Hz. In addition, a secondary peak was identified with a center frequency of 41.70 Hz, an amplitude of 0.064 Hz, and a bandwidth of 5.99 Hz. To enhance the representation of brain oscillatory activity, we applied the FOOOF model individually to each subject for each specific orientation, totaling 60 runs (4 orientations * 15 subjects). We retained up to 15 significant peaks in each fitting, and the distribution plot of these significant center peaks is presented in Fig. 3D. Additionally, we employed a MATLAB kernel density estimation to fit the density function to the histogram, revealing two discernible peaks at 12 and 42 Hz. Remarkably, as evident in Fig. 3C,D, the identified peaks are localized within the alpha and low-gamma frequency bands. This spatial alignment underscores the importance of these peaks, signifying their role in encapsulating distinct oscillatory features within the neural activity under investigation.

**Figure 4:**
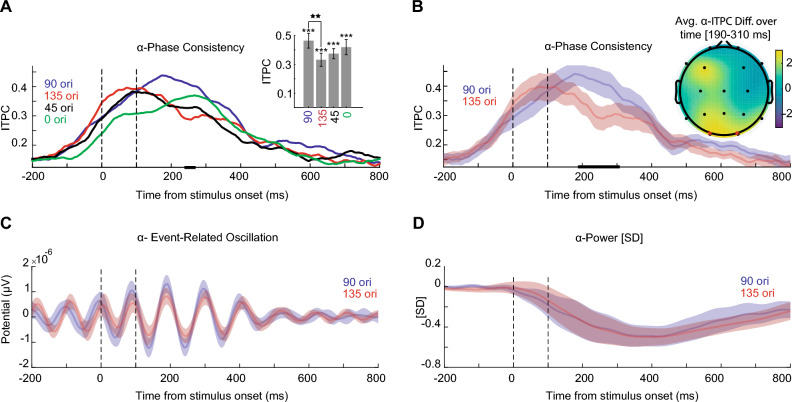
Orientation information in alpha-band frequency. (**A**) Each line shows the average of ITPC values from 15 participants in the occipital area (90^∘^ (blue), 135^∘^ (red), 45^∘^ (black), and 0^∘^ (green)). The dashed line indicates the time of stimuli onset (t = 0 ms) and stimuli offset (t = 100 ms). The Horizontal black lines show the clusters of significant differences between orientations in the time window of 235- to 270 ms (within-subject Friedman, 1000 permutations, participant = 15, cluster-forming threshold p < 0.025, corrected significance threshold p < 0.05). The bar plot indicates averaged ITPC values across the time window of significant periods (235- to 270-ms) of each orientation. Error bars show 95^∘^ confidence intervals calculated across participants (n = 15). The Friedman showed a significant effect on the orientation’s average ITPC value for this time period. Asterisks above horizontal lines indicate significant differences between each pair of orientations (Post-hoc two-sided Wilcoxon tests). n.s: p > 0.05, *p < 0.05, **p < 0.01, ***p < 0.001. (**B**) Same as panel A, the average of ITPC values only for orientation 90^∘^ (blue), 135^∘^ (red) with Error shadings show 95% confidence intervals, calculated across participants (n = 15). The Horizontal lines show clusters of significant differences between orientation 90^∘^ against 135^∘^ (two-sided Wilcoxon tests, 5000 permutations, cluster-forming threshold Z>1.96, corrected significance threshold p < 0.05). Topography shows the associated differences of orientation 90^∘^ against 135^∘^ in alpha z-scored p-values of ITPC (8–12 Hz) in the 190-310 ms window (indicated in the solid line). Star indicates O1 and O2 significant site (5000 permutations, cluster-forming threshold Z > 2.56, corrected significance threshold p < 0.01). (**C**) ERO of alpha-band frequency of 90^∘^ and 135^∘^ orientations, which demonstrated no significant effect (5000 permutations, cluster-forming threshold Z > 1.96). (**D**) The alpha-band power of 90^∘^ and 135^∘^ orientations also showed no significant effect (5000 permutations, cluster-forming threshold Z > 1.96).

In Fig. 4A, each line shows the ITPC average values across 15 subjects in the occipital area for alpha-band frequency (8–12 Hz). We observed a similarity in ITPC value between the 135^∘^ and 45^∘^ as shown, which have the same peak approximately 100-ms after the stimulus onset. The peak of 90^∘^ appeared to be around 200-ms and with a higher value, while the 0^∘^ showed a later peak of roughly 300 -ms and with a lower value. For statistical analysis, at each time point, we performed a within-subject Friedman. For Multiple-comparison-correction we used a cluster-based method (permutation test with n = 1000, cluster-forming threshold p < 0.025, the corrected significance threshold p < 0.05). The Horizontal black lines show the clusters of significant differences between orientations in the time window of 235- to 270-ms, in which we observed that the ITPC value of 90^∘^ orientation increased significantly. Please note that in Fig. 4A, we performed multiple comparisons corrections specifically for time bins. In Supplementary S1 (Figure S1-A), we expanded this correction to a more detailed time-frequency-space analysis. Notably, the significant results within the time window remain consistent with those presented in Fig. 4A. Bar plots show the averaged ITPC values across the time window of 235-270 ms of each orientation: 90^∘^ mean value 0.41 ± 0.04, 135^∘^ mean 0.29 ± 0.04, 45^∘^ mean 0.33 ± 0.03, and 0^∘^ mean 0.37 ± 0.05. The Friedman showed a significant effect on the orientation’s average ITPC value of 235- to 270-ms after stimulus onset ($\chi ^2(3)$ = 7.88, p < 0.05). Post-hoc two-sided Wilcoxon tests showed a statistically significant difference between the 90^∘^ and 135^∘^ (Z = 2.61, p < 0.01, r = 0.67). There was no significant effect on other paired orientations. For clear observation, Fig. 4B demonstrates only the ITPC values of 90^∘^ and 135^∘^ orientations that have large effect sizes. Error shadings show 95% confidence intervals of 15 subjects, and the Horizontal lines show clusters of significant differences between orientation 90^∘^ against 135^∘^ (two-sided Wilcoxon tests, 5000 permutations, cluster-forming threshold Z>1.96, corrected significance threshold p < 0.05).

To reveal the spatial layout of the significant clusters, we visualized the EEG topographies of the Z-value of two-sided Wilcoxon tests orientation 90^∘^ and 135^∘^ for alpha-band ITPC at 190–310 ms after the stimulus onset (Fig. 4B). Star indicates O1 and O2 significant sites (5000 permutations, cluster-forming threshold Z > 2.56, corrected significance threshold p < 0.01). Figure 4C illustrates the ERO of alpha-band frequency of 90^∘^ and 135^∘^ orientations, which showed no significant effect (5000 permutations, cluster-forming threshold Z > 1.96). As depicted in Fig. 4D, the alpha-band power of 90^∘^ and 135^∘^ orientations also had no significant effect.

For a comprehensive visualization of the spatial distribution of the observed effects during periods with significant differences among the four orientations in the alpha band, we have included topoplots for both ITPC and power corresponding to each of the orientations in Supplementary Figure S2.

**Figure 5:**
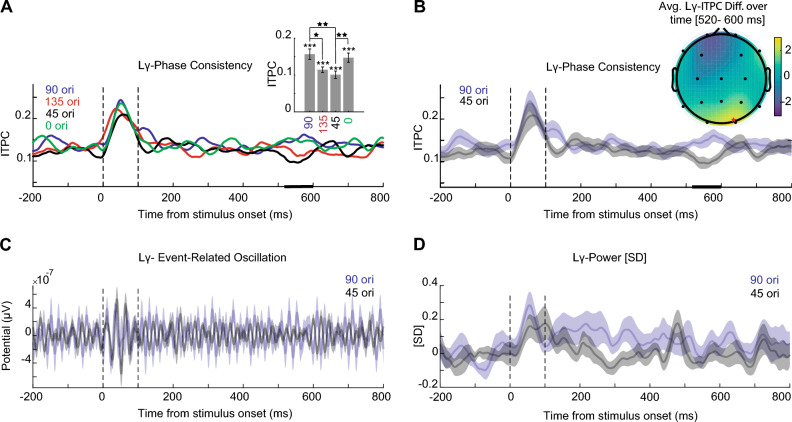
Orientation information in low-gamma-band frequency. (**A**) Each line shows the average of ITPC values from 15 participants in the occipital area (90^∘^ (blue), 135^∘^ (red), 45^∘^ (black), and $0^o$ (green)). The dashed line indicates the time of stimuli onset (t = 0 ms) and stimuli offset (t = 100 ms). The Horizontal black lines show the clusters of significant differences between orientations in the time window of 520- to 600-ms (within-subject Friedman, permutation test with 1000 permutations, participant = 15, cluster-forming threshold p < 0.05, corrected significance threshold p < 0.05). The bar plot illustrates the averaged ITPC values across the time window of significant periods (520 - 600 -ms) of each orientation. Error bars indicate 95^∘^ confidence intervals calculated across participants (n = 15). The Friedman showed a significant effect on the orientation’s average ITPC value for this time period. Asterisks above horizontal lines indicate significant differences between each pair of orientations (Post-hoc two-sided Wilcoxon tests). n.s: p > 0.05 , * p < 0.05, ** p < 0.01, *** p < 0.001. (**B**). Same as panel A, the average of ITPC values only for orientation 90^∘^ (blue), 45^∘^ (black) with Error shadings show 95^∘^ confidence intervals, calculated across participants (n = 15). The Horizontal lines show clusters of significant differences between orientation 90^∘^ against 45^∘^ (two-sided Wilcoxon tests, 5000 permutations, cluster-forming threshold Z > 1.96, the corrected significance threshold p < 0.05). Topography shows the associated differences of orientation 90^∘^ against 45^∘^ in low-gamma z-scored p-values of ITPC (30–50 Hz) in the 520–600 ms window (indicated in the solid line). Star indicates O2 significant site (5000 permutations, cluster-forming threshold Z > 2.56, corrected significance threshold p < 0.01). (**C**) ERO of low-gamma-band frequency for 90^∘^ and 45^∘^ orientations, which showed no significant effect (5000 permutations, cluster-forming threshold Z > 1.96). (**D**) The low-gamma-band power of 90^∘^ and 45^∘^ orientations also showed no significant effect (5000 permutations, cluster-forming threshold Z > 1.96).

The structure of Fig. 5 is the same as Fig. 4 and for low gamma-band frequency (30–50 Hz). We observed no significant effect in the early stage of perception and visual working memory encoding. The Bar plots in Fig. 5A demonstrate the averaged ITPC values across the time window of 520- to 600-ms of each orientation: 90^∘^ mean value 0.15 ± 0.01, 135^∘^ mean 0.12 ± 0.01, 45^∘^ mean 0.10 ± 0.01, and 0^∘^ mean 0.15 ± 0.01. There is a significant difference on the orientation ITPC value of 520- to 600-ms after stimulus onset (Friedman, $\chi ^2(3)$ = 16.3, p < 9.5e-4). Post-hoc two-sided Wilcoxon tests showed a statistically significant difference between the 90^∘^ and 135^∘^ (Z = 1.99, p < 0.05, r = 0.51), as well as on 90^∘^ and 45^∘^ (Z = 2.95, p < 0.003, r = 0.76), and 45^∘^ and 0^∘^ (Z = 2.72, p < 0.006, r = 0.70). There was no significant effect on other paired orientations. Figure 5B demonstrates only the ITPC values of 90^∘^ and 45^∘^ orientations with the largest effect size. The Horizontal black lines show the clusters of significant differences in a late time window of 520- to 600-ms, in which we observed that the ITPC value of 90^∘^ orientation is significantly higher. We visualized the EEG topographies of the Z-value for two-sided Wilcoxon tests of orientations 90^∘^ and 45^∘^ in low-gamma-band ITPC at 520- to 600-ms post-stimulus onset (Fig. 5B). The star indicates the O2 electrode significant site (5000 permutations, cluster-forming threshold Z > 2.56, corrected significance threshold p < 0.01). Figure 5C illustrates the ERO of low-gamma-band frequency of 90^∘^ and 45^∘^ orientations, which showed no significant effect (5000 permutations, cluster-forming threshold Z>1.96). As depicted in Fig. 5D, the low-gamma-band power of 90^∘^ and 45^∘^ orientations also had no significant effect. It is notable that in Fig. 5A, we performed multiple comparisons corrections specifically for time bins. In Supplementary S1 (Fig. S1-B), we expanded this correction to a more detailed time-frequency-space analysis. Notably, the significant results within the time window remain consistent with those presented in Fig. 5A.

Again, for the spatial distribution of the observed effects in the low-gamma band, we have included topoplots for both ITPC and power corresponding to each of the orientations in Supplementary Figure S3.

## Discussion

We investigated whether the maintained information in VWM about the specific orientation of the to-be-remembered object is available in the alpha and low-gamma-band activity in EEG measurements. Our simple experimental design showed that the orientation information of the four Gabor gratings is available in the trial phase consistency of alpha and low-gamma-band oscillations.

The observed alpha suppression aligns with memory-related cognitive processes during a crucial period. This sustained suppression in the alpha range corresponds to active engagement in Working Memory tasks, indicating a reduction in alpha/beta power. This supports the notion that these dynamics reflect neural mechanisms involved in forming and maintaining active representations within Working Memory.

During the delay period of VWM tasks, recent studies suggest that the EEG activity encloses the information about the content of actual VWM representations^21,42^. However, the evidence is based on the decoding methods, which used low-frequency ERP (under 6Hz), power-band analysis of scalp EEG distributions, or the raw EEG data of a noticeable number of electrodes in the occi-parietal area of the brain. Furthermore, they found little or no evidence of maintained orientation information in the alpha-band or any frequency band oscillations besides the low-frequency sustained activity (under 6Hz). In contrast to the previous studies, we showed that with only two electrodes in the occipital area, the information of different orientations is distinctive in the phase of alpha-band activity.

Although recent studies showed the representation of information mainly occurs in the early stage of sensory visual encoding in alpha-band, we illustrated that the orientation information emerged in the phase value of low gamma-band oscillation in late periods. This finding may reflect memory traces^43,44^, which tell us memory traces of oblique orientation deficient activation relative to the cardinal axis.

Examining the observed differences in ITPC values in the occipital region, in the early phase in the alpha frequency range and the late phase in the low gamma frequency range, initiates research on the potential causal factors underlying the response variability. An interesting aspect of this research lies in the subtle response of the directional stimulus, especially the divergence between the cardinal and oblique stimuli, in the low alpha and gamma frequency bands. This difference in response patterns may reflect complex neural processing mechanisms, suggesting that differential engagement of these frequency bands plays a pivotal role in shaping cortical dynamics associated with orientation-specific stimuli. Elucidation of such subtleties is of great importance in understanding the complex interaction between neural oscillations and stimulus-specific processing and contributes to a more precise understanding of cognitive processes in the occipital cortex.

Also, the ITPC values during the period when there was a significant difference between different orientations (Figs. 4A-right and–5A-right) are consistent with findings that show the distribution of orientation selectivity in the several mammals and human primary visual cortex, are oriented around cardinal orientations relative to oblique orientations^45–47^. This result can be expected, because the Distribution of orientation selectivity cardinal axis is greater than in the oblique, and this causes more phase consistency and synchrony in the cardinal directions than in the oblique, and this can be seen in ITPC value. the important point is that this effect appeared in in the early phase in the alpha frequency range and the late phase in the low gamma frequency range.

In conclusion, the present study attempted to find whether any other effective measurements could provide sufficient information about orientation representations in VWM when the ERP and power failed to do so. Moreover, it is essential to find the mechanism of visual information distribution that could help us find more straightforward methods for decoding the specific information maintained in VWM. Therefore, we showed that the orientation information of the presented stimuli is distinguishable in the phase consistency of trials in alpha and low-gamma-band activity of the occipital regions. Although it is not possible to use ITPC analysis directly in decoding methods, revealing the existence of this information in the phase values of alpha-band suggests that future studies could develop strategies that involve phase (e.g., using a phase-amplitude coupling) to reach the expected results more reliably.

### Supplementary Information

Supplementary Information.

## Data Availability

The Data and code may be provided to interested researchers upon reasonable request to the corresponding author.
