# Supplementary material for: Discriminating orientation information with phase consistency in alpha and low-gamma frequency bands: an EEG study
Source: Sci Rep. 2024 May 25;14:12007. doi: 10.1038/s41598-024-62934-y (PMC11127946; doi:10.1038/s41598-024-62934-y)
Supplement: Supplementary file 1 — Supplementary Information. [file 41598_2024_62934_MOESM1_ESM.pdf]

Supplementary Information

Discriminating Orientation Information with Phase Consistency in Alpha and Beta Frequency Bands: An EEG Study

Alireza Khadir, Shamim Sasani Ghamsari, Samaneh Badri, Borhan Beigzadeh*

Biomechatronics and Cognitive Engineering Research Lab, School of Mechanical Engineering, Iran University of Science and Technology, Tehran, Iran

* Correspondence: b_beigzadeh@iust.ac.ir, Tel: +98-21-77240094

Supplementary S1

To enhance the rigor of our results in the alpha and low-gamma bands, we conducted a comprehensive time-frequency-space analysis. Our main text focused on multiple comparison correction for time bins. Here, we demonstrate that our results align with a more stringent correction method.

To achieve this, we refined our approach by narrowing the spatial dimension. According to our classification and our result which was in the occipital region, we have a neighborhood and that is the parietal region. Therefore, we changed the significance level value of α from 0.05 to 0.025 with strict Bonferroni correction. This change of the threshold value ensures that we consider the spatial correction.

This refinement allowed us to address the sensitivity of reported p-values across time and frequency, transitioning to a two-dimensional representation with time on the x-axis and frequency on the y-axis. We implemented a whole-subject permutation test using Friedman's core and applied cluster-based multiple correction. Notably, this comprehensive analysis identified two significant regions, consistent with the one-dimensional time-bin analysis. While the intricacies of these findings may be challenging to convey in the main text, we have presented them in Figure S1.

These additional analyses contribute to the robustness and consistency of our findings, reinforcing the validity of our results in alpha and low-gamma frequency bands.

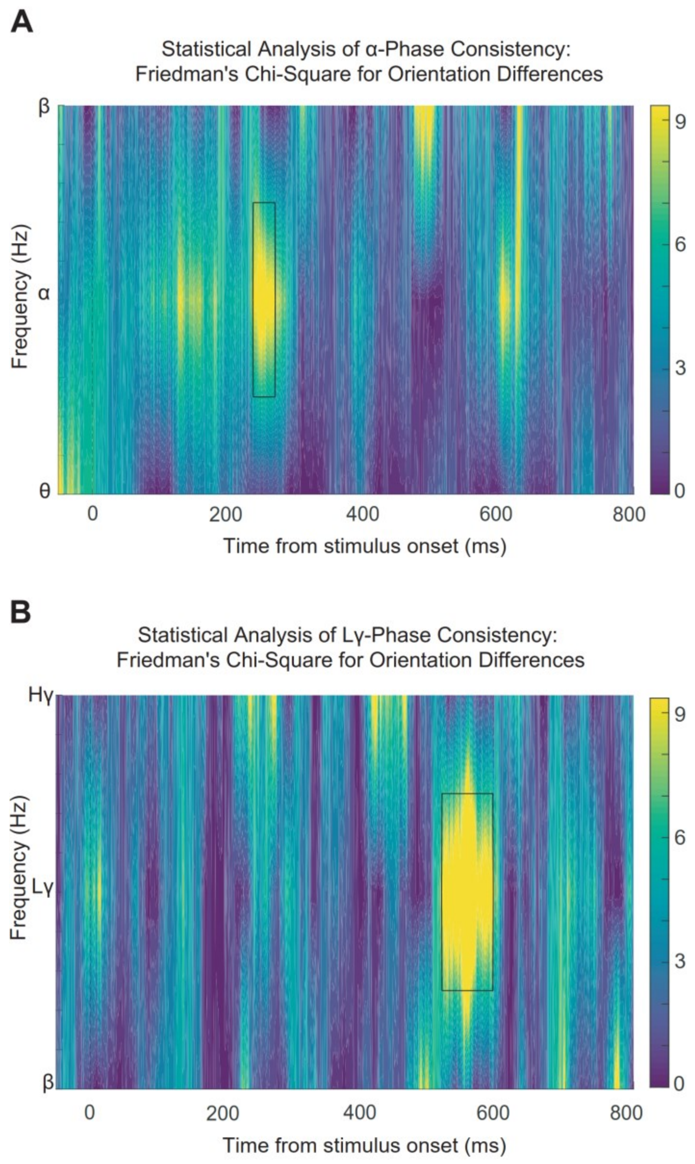

Figure S1 - A) Time-frequency analysis of Friedman's Chi-Square values for Orientation Differences in ITPC in the occipital area in the alpha-band (8-12 Hz). The highlighted cluster in the 230-270 ms after stimulus onset indicates a significant ITPC difference (permutation test with 1000 permutations, participants = 15, cluster-forming threshold $p < 0.025$, corrected significance level $p < 0.05$). **B)** Same as A for the low gamma-band (30-50 Hz). The highlighted cluster in the 520-600 ms after stimulus onset indicates a significant ITPC difference (permutation test with 1000 permutations, participants = 15, cluster-forming threshold $p < 0.025$, corrected significance level $p < 0.05$).

Supplementary S2 & S3

In Figures 4 and 5 within the main paper, which correspond to the time periods exhibiting significant differences among the four orientations (alpha and low-gamma, respectively), we have included topoplots for both ITPC and power for each of the four orientations. This provides a comprehensive visualization of the spatial distribution of the observed effects. Furthermore, to ensure accessibility and transparency, we have included these topoplots in Figure S2 and Figure S3 for readers to examine in detail.

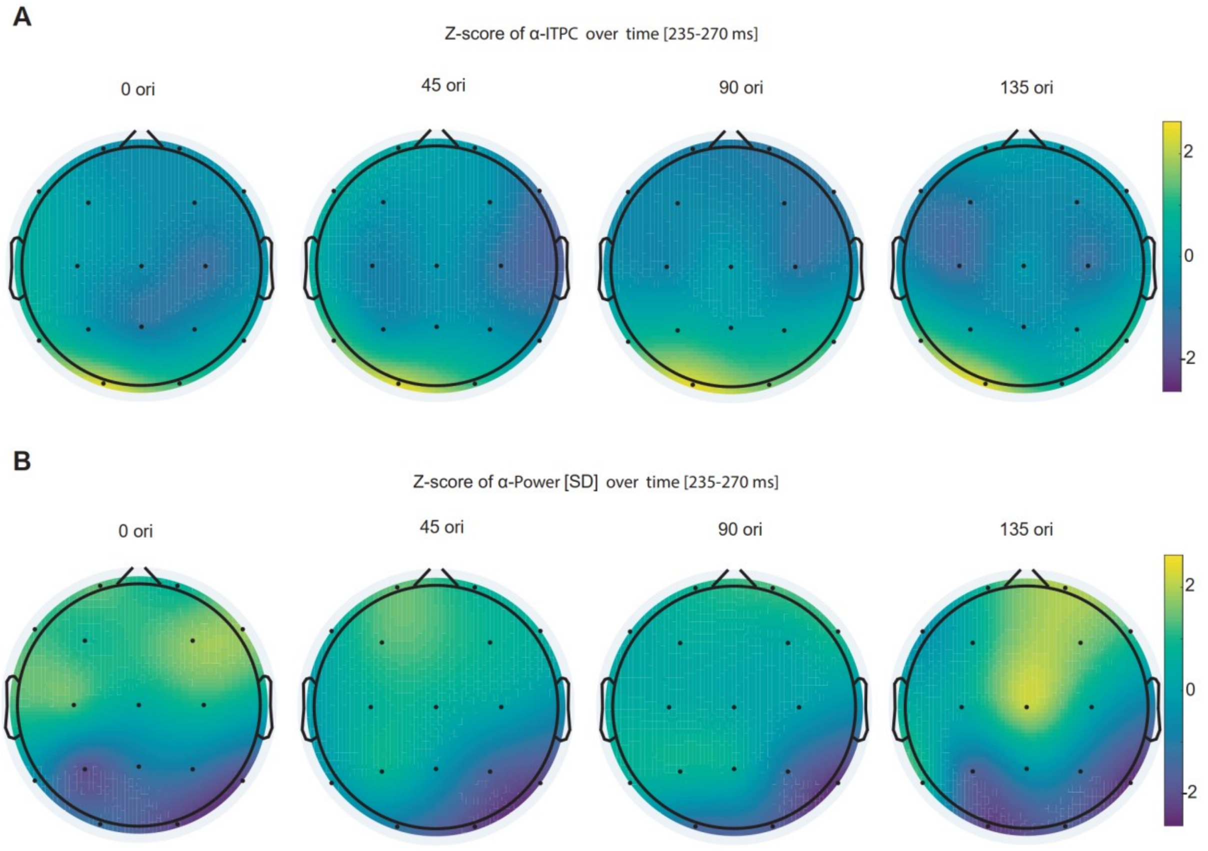

Figure S2 - Top: Topography shows the z-scored of alpha ITPC (8-12 Hz) in the 235-270 ms window after stimulus onset for four orientations. Bottom: Same as above for z-scored of alpha power

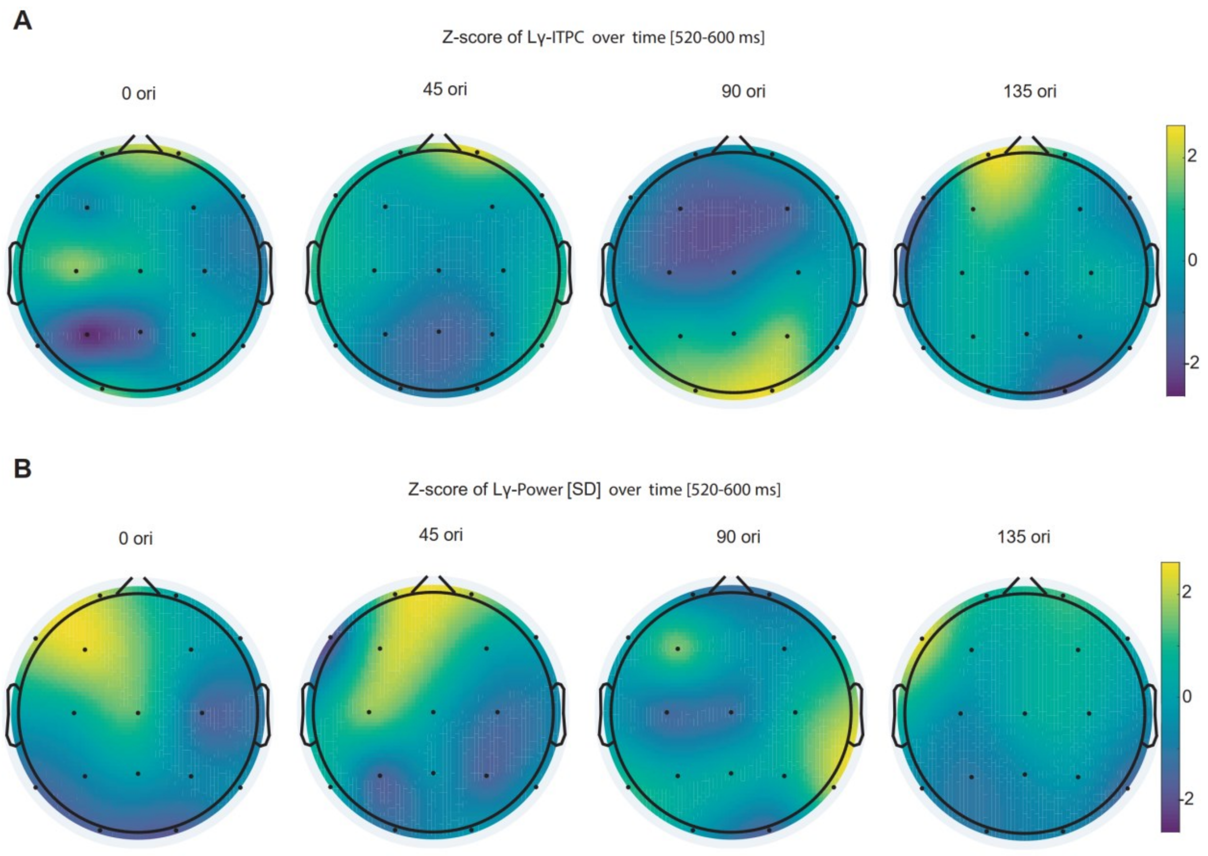

Figure S3 - Top: Topography shows the z-scored of low-gamma ITPC (30-50 Hz) in the 520-600 ms window after stimulus onset for four orientations. Bottom: Same as above for z-scored of low-gamma power
